# HPV status shapes T-cell immunoprofiles in oesophageal adenocarcinoma: high regulatory T cell infiltration predicts poor prognosis

**DOI:** 10.1186/s12967-025-07482-3

**Published:** 2025-11-27

**Authors:** Subin Wui, Rehana V. Hewavisenti, Mohammad Rabiei, Anthony D. Kelleher, Mahtab Farzin, Shanmugarajah Rajendra, Sarah C. Sasson

**Affiliations:** 1 Immunovirology and Pathogenesis Program, The Kirby Institute, The University of New South Wales, Kensington, Sydney, NSW 2033 Australia; 2 Faculty of Medicine and Health, School of Clinical Medicine, University of New South Wales, Kensington, Sydney, NSW Australia; 3 Gastro-Intestinal Viral Oncology Group, Ingham Institute for Applied Medical Research, 1 Campbell Street Liverpool, Sydney, NSW 2170 Australia; 4 South Western Sydney Clinical School, University of New South Wales, Kensington, Sydney, NSW Australia; 5 Department of Anatomical Pathology, Liverpool Hospital, Liverpool, Sydney, NSW Australia; 6 Department of Pathology, Campbelltown Hospital, Campbelltown, Sydney, NSW Australia; 7 Western Sydney University, Campbelltown, Sydney, NSW Australia; 8 Department of Gastroenterology & Hepatology, Bankstown-Lidcombe Hospital, Bankstown, Sydney, NSW Australia

**Keywords:** Human papillomavirus, Oesophageal adenocarcinoma, Tumour microenvironment, Regulatory T-cells, CD8+ T-cells, Biomarker

## Abstract

**Background:**

Oesophageal adenocarcinoma (OAC) outcomes remain poor, with a 5-year survival rate of ~20%. Recent evidence suggests that human papillomavirus (HPV) is associated with ~35% of OAC. HPV-positive OAC patients have higher survivorship compared to HPV-negative patients, however current management remains uniform regardless of HPV status. Although the tumour microenvironment (TME), particularly intra-tumoural T-cell infiltration, is associated with improved outcomes in numerous cancers, characterisation of T-cell subsets in HPV-driven and HPV-independent OAC have not been explored.

**Methods:**

Differences in T-cell subsets within OAC biopsies of HPV-positive (*n* = 15) and HPV-negative (*n* = 22) were studied by multiplex immunofluorescence microscopy, in a retrospective cross-sectional study. Statistical analyses were conducted to assess differences in T-cell quantification, and to assess the correlation with patient prognosis.

**Results:**

Increased densities of FoxP3+ regulatory T-cells (Treg) and reduced CD8+:Treg ratios were evident in HPV-negative OAC compared to HPV-positive OAC. Other CD4+ and CD8+ subset densities were comparable. Univariate analyses demonstrated shorter overall survival (OS) in patients with high Treg proportions and low CD8+:Treg ratios. Multivariate analyses demonstrated a low total CD8+:Treg ratio to be independently associated with poor prognosis (HR 3.06; 95% CI 1.05–10.27; *p* = 0.050), along with metastasis and absence of HPV infection.

**Conclusion:**

We report novel evidence of an immunoregulatory TME in HPV-negative OAC, as indicated by high Treg proportions and low CD8+:Treg ratios. Future work may lead to improved risk stratification by the presence or absence of HPV, as well as utilising the CD8+:Treg ratio in the local TME as a predictive biomarker. Additionally, a more in-depth understanding of the TME would assist in the development of novel targeted immunotherapies, for example by modulating Tregs or CD8+ T-cells.

**Supplementary information:**

The online version contains supplementary material available at 10.1186/s12967-025-07482-3.

## Background

Oesophageal cancer, remains a significant global health challenge, ranking as the seventh leading cause of cancer-related mortality worldwide, and claiming 445,129 deaths in 2022 alone. Despite advances in treatment, the prognosis remains poor, with a 5-year survival rate under 20% [1]. There are two histological subtypes: squamous cell carcinoma (SCC), which accounts for ~85% of cases, and adenocarcinoma, where the incidence is rising up to 8.1% per year across Western countries [2]. Oesophageal adenocarcinoma (OAC) has a glandular morphology and is classically understood to develop through the multistep Barrett’s metaplasia-dysplasia-adenocarcinoma sequence, driven by gastro-oesophageal reflux disease (GORD) [3]. However, this paradigm is being challenged, as GORD rates are declining in high socio-demographic index regions, suggesting additional etiological factors [4]. Recent evidence suggests that up to 35% of OAC is associated with human papillomavirus (HPV), particularly the high-risk oncogenic strains HPV-16 and HPV-18 [5]. This is supported by molecular evidence identifying HPV DNA and mRNA in OAC tissues [6]. Given the immunogenic nature of HPV and its oncogenic potential via early (E) oncogenes and late (L) genes for viral assembly, integration and replication, HPV-positive OAC appears to represent a genetically distinct entity [7], opening new avenues for understanding pathogenesis and therapeutic targeting.

HPV-positive OAC follows the survival trend of other HPV-driven malignancies such as cervical cancer [8] and head and neck SCC (HNSCC) [9], demonstrating superior disease-free survival (DFS) compared to HPV-negative counterparts [10]. However, OAC is currently treated in a standardised manner regardless of HPV status, highlighting inefficient use of healthcare resources and the future potential for developing more specific treatment strategies. This emphasises the need for improved understanding of the divergent pathogenesis and host response between HPV-positive and HPV-negative OAC, to enable more accurate risk stratification, guide treatment de-escalation and advance personalised therapies.

Cytotoxic CD8+ T-cells play a crucial role in anti-viral and anti-tumour immunity, and their presence in the tumour microenvironment (TME) is correlated with favourable prognosis in a range of cancers including HPV-driven cervical cancer [11] and HNSCC [12]. Furthermore, T-cells are direct targets of immune checkpoint inhibitor (ICI) therapies and serve as predictive biomarkers for treatment response [13]. Concordantly in OAC, prognosis is linked to local T-cell populations, with higher CD4+, CD8+, CD103+ CD8+ tissue-resident memory (CD8+ T_RM_), and CD45RO+ memory T-cells associated with DFS and overall survival (OS) [14, 15].

In HNSCC, HPV-positive TME demonstrated higher concentrations of CD4+ helper T-cells, cytotoxic CD8+ T-cells, CD103+ CD8+ tissue-resident memory (CD8+ T_RM_) T-cells and CD45RO+ memory T-cells compared to HPV-negative counterparts, supporting the hypothesis that antigenicity of HPV may augment anti-tumour immunity [16, 17]. To date, unlike HNSCC, variation of T cells within the TME of HPV-positive versus HPV-negative OAC has not been explored. Therefore, this study aimed to quantify T-cell subsets in the TME of both HPV-associated and HPV-negative OAC and evaluate the relationship with OS to identify potential predictive biomarkers for OAC.

## Methods

### Patient samples

This retrospective cross-sectional study followed the ‘Strengthening the Reporting of Observational Studies in Epidemiology’ guidelines. Histologically-confirmed human Siewert Class 1 OAC biopsies were studied, defined as distal oesophageal tumours with the epicentre located 1 to 5 cm above the gastroesophageal junction (*n* = 37; Table 1). All samples were formalin-fixed paraffin-embedded (FFPE) and collected between the years 2012 to 2019. These pre-treatment specimens were from an existing tissue biobank at a tertiary referral centre (Liverpool Hospital, NSW, Australia). Eligibility criteria for study inclusion and exclusion have been previously outlined [7]. Demographic and clinical data were obtained from a prospectively maintained database.

**Table 1:** Clinicopathological characteristics of the patient cohort

	All Patients		HPV-negative		HPV-positive	
	n	(%)	n	(%)	n	(%)
**Age** (years)						
Median (Range)	78 (50–92)		76 (50–90)		79 (53–92)	
**Sex**						
Male	29	(78)	16	(73)	13	(87)
Female	8	(22)	6	(27)	2	(13)
**Stage**^**a**^						
I	17	(46)	7	(32)	10	(67)
II	6	(16)	5	(23)	1	(7)
III	6	(16)	4	(18)	2	(13)
IV	8	(22)	6	(27)	2	(13)
**T stage**^**a**^						
Low (T1–T2)	21	(57)	10	(46)	11	(73)
High (T3–T4)	14	(38)	10	(46)	4	(27)
Data Not Available	2	(5)	2	(9)	0	(0)
**N stage**^**a**^						
Negative (N0)	23	(62)	11	(50)	12	(80)
Positive (N1–N2)	14	(38)	11	(50)	3	(20)
**M stage**^**a**^						
Negative (M0)	27	(73)	15	(68)	12	(80)
Positive (M1)	10	(27)	7	(32)	3	(20)
**Death**						
No	15	(41)	7	(32)	8	(53)
Yes	22	(60)	15	(68)	7	(47)
**HPV gene**						
E					7	(47)
L					8	(53)
**HPV viral load**						
Median (Range)					14.98 (0.06–115.03)	

^**a**^AJCC 7^th^ edition staging system(22). T, tumour. N, lymph node. M, metastasis

### HPV testing

HPV status on patient samples was determined by nested multiplex polymerase chain reaction (PCR) assays as previously published [18, 19]. HPV viral load was determined by real-time PCR measurement of E6 and L1 gene copy numbers [20]. Primer sequences for HPV-16, HPV-18, and the human albumin gene are detailed Supplementary Table S1. HPV-positivity was defined by the positive expression of E or L genes from HPV-16 or HPV-18 and a detectable viral load. Fifteen samples were HPV-positive and twenty-two were HPV-negative. Authors conducting image analysis were blinded to the HPV status.

To minimise PCR contamination, all steps were physically conducted in separate rooms, including reaction preparation, template handling, nested PCR setup and post PCR analysis. Workspaces, including the DNA-free PCR hood, was routinely decontaminated using UV irradiation prior to each run. To monitor for systematic contamination of PCR reagents, positive controls (HPV-16 positive cervical cancer tissue) and negative controls (deionised water and PCR master mix without template) were included in each stage. Each PCR assay was performed in triplicate to ensure reproducibility. Additionally, to prevent cross-contamination between FFPE tissue samples, the microtome blade was replaced between each patient block.

### Multiplex immunofluorescence (mIF) staining

FFPE tissue blocks were sectioned at 4 μm thickness and stained using Opal 7-Color Automation IHC Kit (Akoya Biosciences, #NEL821001KT). Briefly, FFPE slides were baked at 58 °C for 1 hour, deparaffinised in xylene (2x5 minutes) and rehydrated in an ethanol gradient from 100% (3x1 minute) to 70% (1 minute). Epitope retrieval was performed for 5 minutes using citrate-based pH 6 (Dako, #K8005) or EDTA-based pH 9 buffer (Dako, #8004) in the NxGen Decloaking chamber (BioCare Medical) at 110 °C. Multiplex immunofluorescence (mIF) microscopy was performed on the BOND RX automated immunostainer (Leica Biosystems). Incubation of primary antibody targeting the protein of interest (e.g. CD4) was carried out for 30 or 60 minutes at room temperature, followed by the application of horseradish peroxidase (HRP) secondary antibody, and a corresponding Opal fluorophore. Slides were washed in tris-buffered saline (Dako, #K8007) between incubation steps. Epitope retrieval was performed again using citrate-based pH 6 (Leica Biosystems, #AR9961) and EDTA-based pH 9 buffer (Leica Biosystems, #AR9640). After the staining of all fluorophores was complete, DAPI was counterstained for 5 minutes to detect the cell nuclei and mounted with Prolong Gold anti-fade mounting medium (Thermo Fisher Scientific). Tissue slides were cured for 24 hours at room temperature in the dark until imaging. Primary antibodies used in the panel were PanCK (Thermo Fisher Scientific Cat# A500-019A, RRID:AB_2773816) to visualise epithelial cells, and T-cell markers of CD4 (Abcam Cat# ab133616, RRID:AB_2750883), CD8 (Thermo Fisher Scientific Cat# MA5-13473, RRID:AB_11000353), FoxP3 (Abcam Cat# ab20034, RRID:AB_445284) and CD103 (Abcam Cat# ab129202, RRID:AB_11142856). A representative image of the mIF panel is outlined in Supplementary Figure S1.

### Haematoxylin and Eosin (H&E) staining

We performed H&E staining on all serial sections to cross-validate tumour regions, ensuring that PanCK+ cells identified in mIF were exclusively tumour cells. An experienced anatomical pathologist confirmed and annotated the tumour regions as regions of interest (ROI).

### Image analysis

**Fig. 1:**
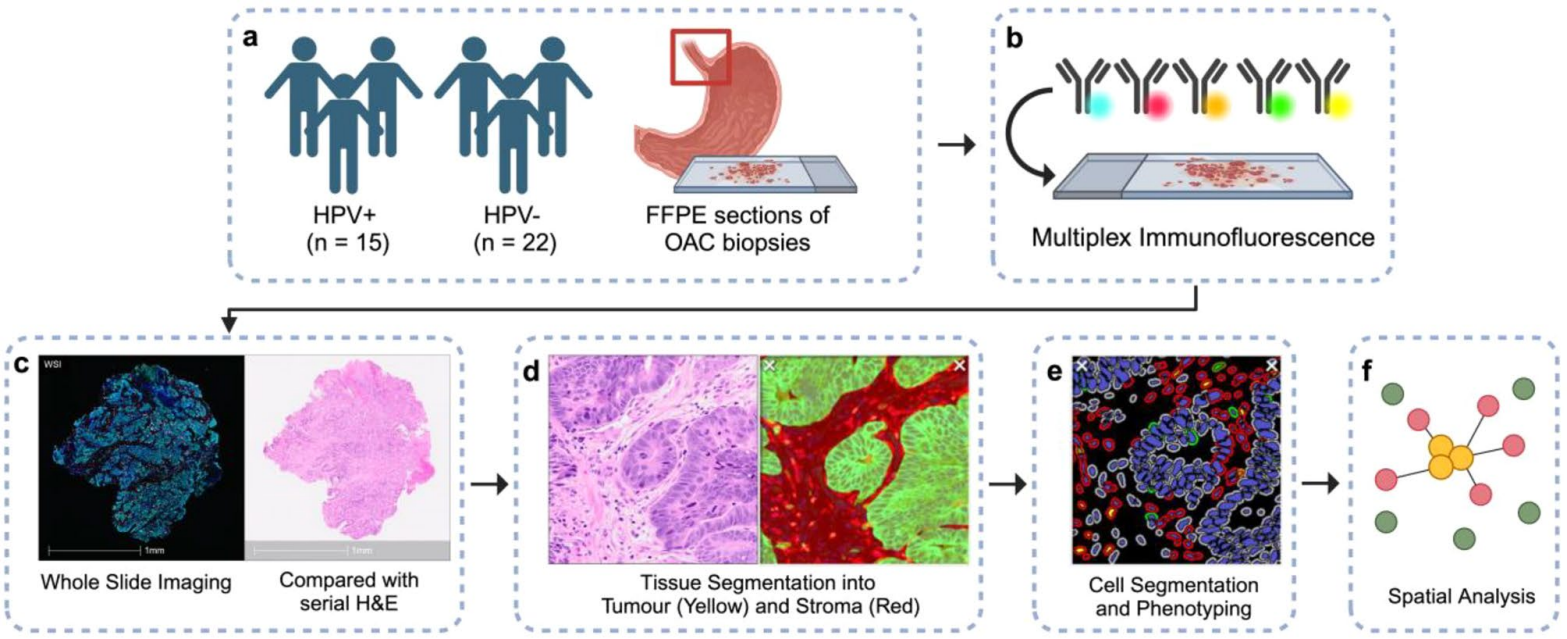
Schematic diagram of the analysis pipeline. We studied (**a**) formalin-fixed paraffin-embedded OAC tissues by conducting (**b**) mIF. (**c**) The whole slide was imaged and compared with H&E to determine the roi. (**d**) Tissue segmentation was guided by H&E and panCK positivity. (**e**) T-cell subsets were identified, followed by (**f**) spatial analysis around tumour cells (yellow). Created with Biorender

The workflow following the mIF of 37 OAC tissues is outlined in Fig. 1. Whole sections of mIF stained slides were scanned at 20x magnification using the Vectra Polaris Automated Quantitative Pathology Imaging System (Akoya Biosciences; RRID:SCR_025508; Fig. 1c). Multispectral images were then unmixed using inForm v.2.5 (Akoya Biosciences; RRID:SCR_019155). Individual images were loaded onto the HALO^®^ v3.6 (Indica Labs; RRID:SCR_018350), an image analysis software for quantitative and qualitative analyses. The ROI included tumour tissue identified by the pathologist (Fig. 1c) and excluded any large tears from tissue processing. Within HALO^®^, tissue was segmented into tumour and stromal regions by creating annotations and training the MiniNet Classifier, a deep-learning neural network algorithm (Fig. 1d). Tumour region was defined as the PanCK-positive area and the remainder of the tissue was annotated as stroma. The performance of tissue segmentation by the MiniNet Classifier was verified on random areas of different slides with varying morphology. Cell segmentation was completed by adding annotations of the nuclei based on DAPI staining and training the in-built Nuclei Seg Classifier.

Using the HALO^®^ Highplex-Fluorescent module v.4.2 (Indica labs; RRID:SCR_018350), cytoplasm and nuclear signal intensity thresholds for each single-colour fluorophore were set, to identify true positive staining while eliminating background stains. This was reviewed across multiple regions within the tissue by cross-checking the counts of markers by visualisation in real-time tuning (Fig. 1e). T-cells subsets of interest were defined using CD8, CD4, CD103, and FoxP3 fluorescent markers (Supplementary Figure S2). CD103+ T-cells indicate tissue-resident T-cells for long-term tissue residence [21], while CD103- T-cells represented classical T-cell subset that has recently infiltrated the tissue from peripheral blood. Classical and tissue-resident CD4+ T-cells were further divided into regulatory T-cells (Treg) by FoxP3 positive staining.

The optimised MiniNet classifier (tissue segmentation), NucleiSeg Classifier (cell segmentation), and Highplex-Fluorescent module (phenotyping) were applied to the whole ROI of all tissues. T-cell subsets were quantified as cell density (cell count per total area) within the total area, intratumoural and stromal regions. Spatial distribution of T-cells around tumour (PanCK+) cells was also investigated using a proximity analysis module in HALO^®^ (Indica Labs; RRID:SCR_018350), calculating the average distance between each T-cell subset and tumour cell, and the percentage of each subset within 50 μm radii of tumour cells (Fig. 1f).

### Statistical analysis

All statistical analyses were conducted on GraphPad Prism v10.2 (RRID:SCR_002798), with statistical significance set at *p* < 0.05. Unpaired non-parametric Mann-Whitney U tests (two-tailed) were used to compare T-cell parameters between HPV-positive and HPV-negative cohorts. To assess the correlations with patient outcomes, patients were grouped into high vs low T-cell variable cohorts, with high defined as ≥median. The OS of dichotomised cohorts were displayed on Kaplan Meier survival curves and the significance of the difference was evaluated by the log-rank Mantel-cox test. Multivariate Cox proportional hazards modelling was used to determine the independent hazard ratio (HR) with respect to OS, defined as the time from biopsy to death or to the last follow-up date. The cut-off date for patient outcome data was 31st July 2024, with data analysis from July to November 2024.

## Results

### Patient characteristics

The characteristics of participants (*n* = 37) are summarised in Table 1. The median age was 78 years (range 50–92 years), with 78% being male. Median follow-up length for OS was 94 months (range 2–410 months), with 22 deaths during the study period. Of the HPV-positive cases, there was an even distribution of HPV E and L gene identified in the nested PCR assays, with a median viral load of 14.98 copies per 1000 cells (Supplementary Table S2). According to the TNM classification of the 7^th^ edition of the American Joint Committee on Cancer (AJCC) for cancers of the gastro-oesophageal junction [22], there were 17 cases classified as stage I, 6 cases as stage II, 6 cases as stage III and 8 cases as stage IV. Tumour stage was grouped into low (T1–T2) and high (T3–T4) groups due to a limited sample size when categorised by individual stages (T1–T4). 38% had lymph node positive OAC, and 27% had distant metastases.

### Treg (FoxP3+ CD103- CD4+) density is higher in HPV-negative OAC

**Fig. 2:**
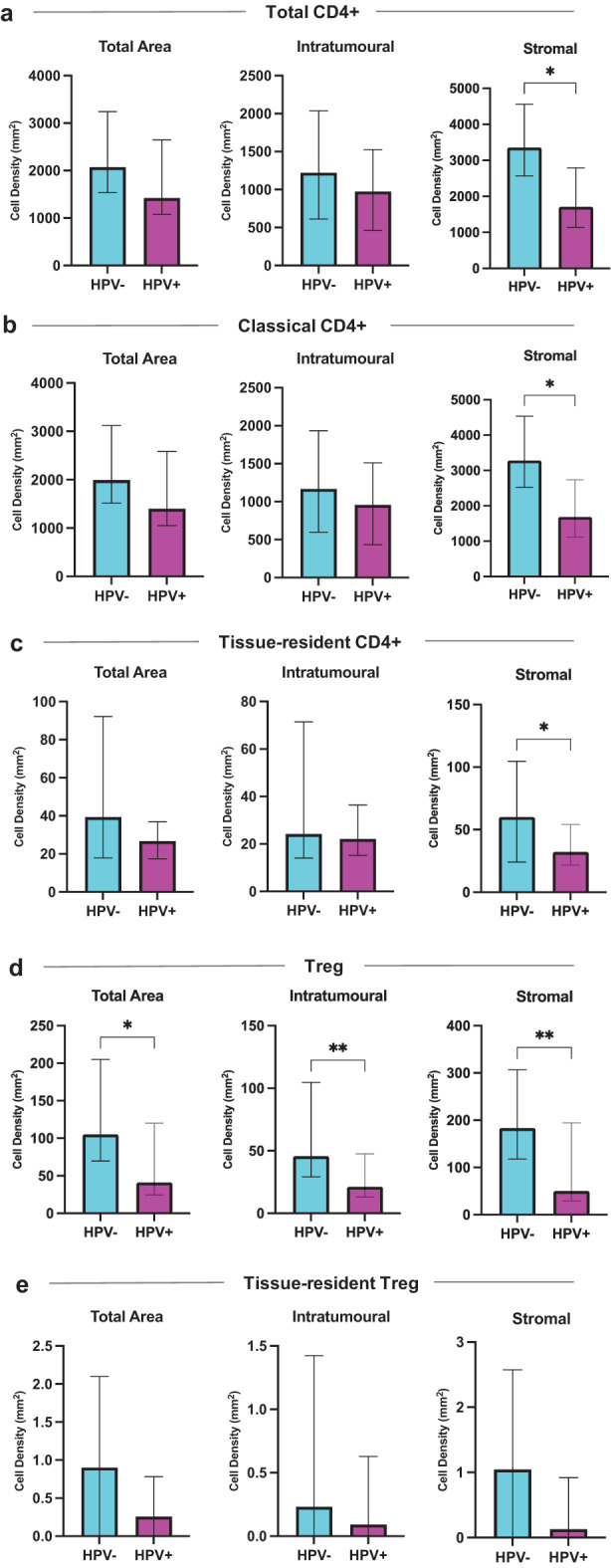
CD4+ T-cell densities in HPV-negative and HPV-positive OAC. (**a**) Total CD4+ T-cells. (**b**) Classical CD4+ T-cells. (**c**) Tissue-resident CD4+ T-cells. (**d**) Treg (**e**) Tissue-resident Treg. Unpaired nonparametric mann-whitney U tests were performed on (**a**)-(**e**). Results are presented as median with 95% ci; non-significant *p* > 0.05; *p < 0.05; **p < 0.01

**Fig. 3:**
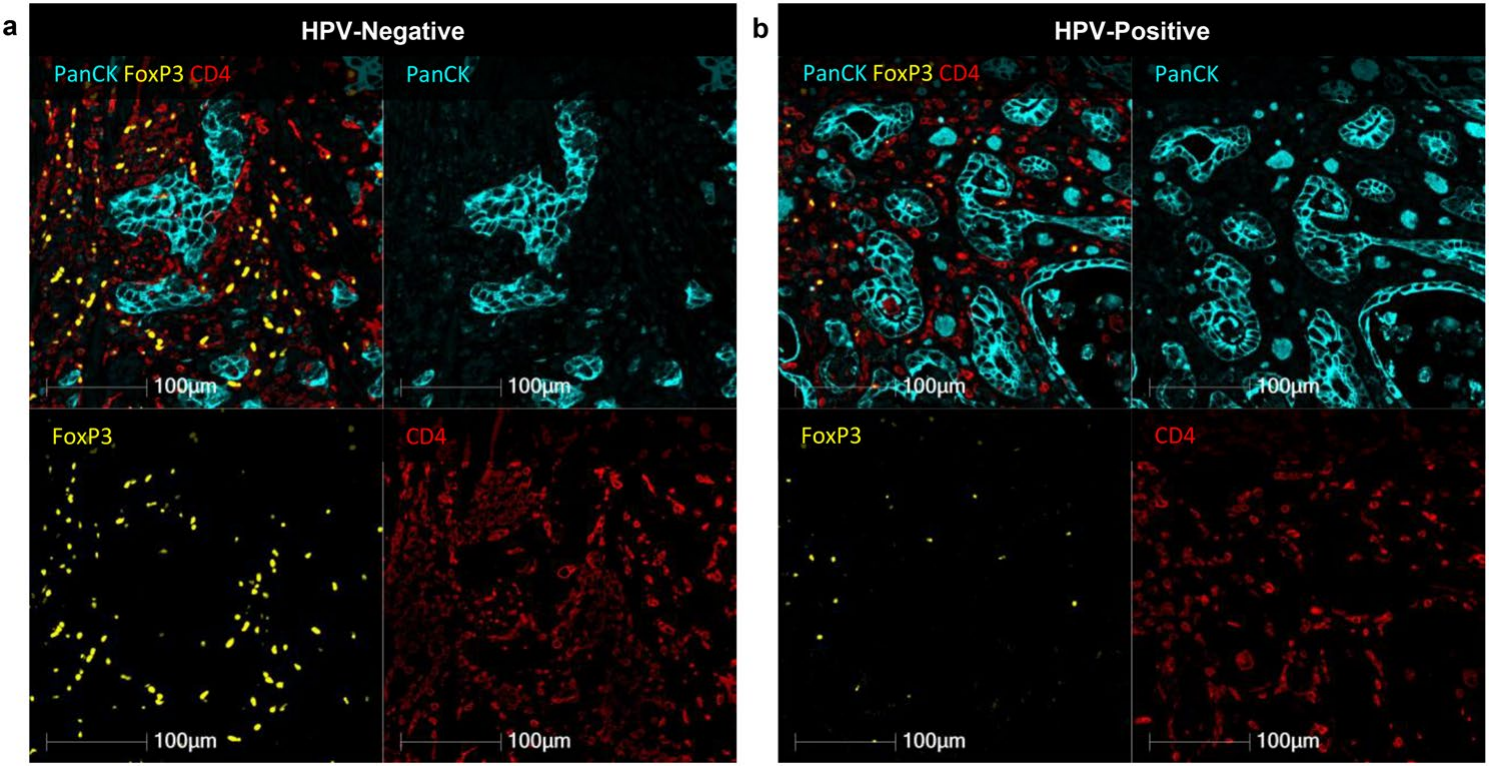
Representative multiplex immunofluorescence images of FoxP3+ tregs in HPV-negative and HPV-positive OAC (**a**) HPV-negative and (**b**) HPV-positive OAC are shown demonstrating higher infiltration of CD4+ (red) T-cells expressing FoxP3 (yellow) in HPV-negative compared to HPV-positive OAC. PanCK (cyan) identifies tumour cells (PanCK; cyan). Scale bar 100 μm. Representative images are zoomed areas within a 20x objective

CD4+ T-cell numbers varied between HPV-positive and HPV-negative OAC (Fig. 2). Specifically, total CD4+ T-cell density in the stroma was higher in HPV-negative OAC compared to HPV-positive OAC (3354 vs 1706/mm2; *p* = 0.02; Fig. 2a). Similarly, both classical CD4+ (CD103- CD4+; *p* = 0.02; Fig. 2b) and tissue-resident CD4+ T-cell (CD103+ CD4+; *p* = 0.03; Fig. 2c) counts were higher in the HPV-negative stroma. No significant differences were observed in the total area or intratumoural region for total, classical and tissue-resident CD4+ T cells (Fig. 2a–c). Strikingly, there were increased proportions of Tregs (FoxP3+ CD103- CD4+) in HPV-negative OAC compared to HPV-positive OAC (Fig. 3). This difference was observed across all histological areas assessed, i.e. total area (105 vs 41/mm2; *p* = 0.01; Fig. 2d), intratumoural (46 vs 21/mm2; *p* = 0.008; Fig. 2d) and stromal regions (183 vs 50/mm2; *p* = 0.009; Fig. 2d). Conversely, there was no significant difference in the tissue-resident Tregs (FoxP3+ CD103+ CD4+; Fig. 2e). Total CD8+, classical CD8+ (CD103- CD8+) and CD8+ T_RM_ (CD103+ CD8+) T-cell densities were comparable between HPV-positive and HPV-negative OAC in all histological areas assessed (Supplementary Figure S3).

### Lower CD8+ T-cell to Treg ratios in HPV-negative OAC

**Fig. 4:**
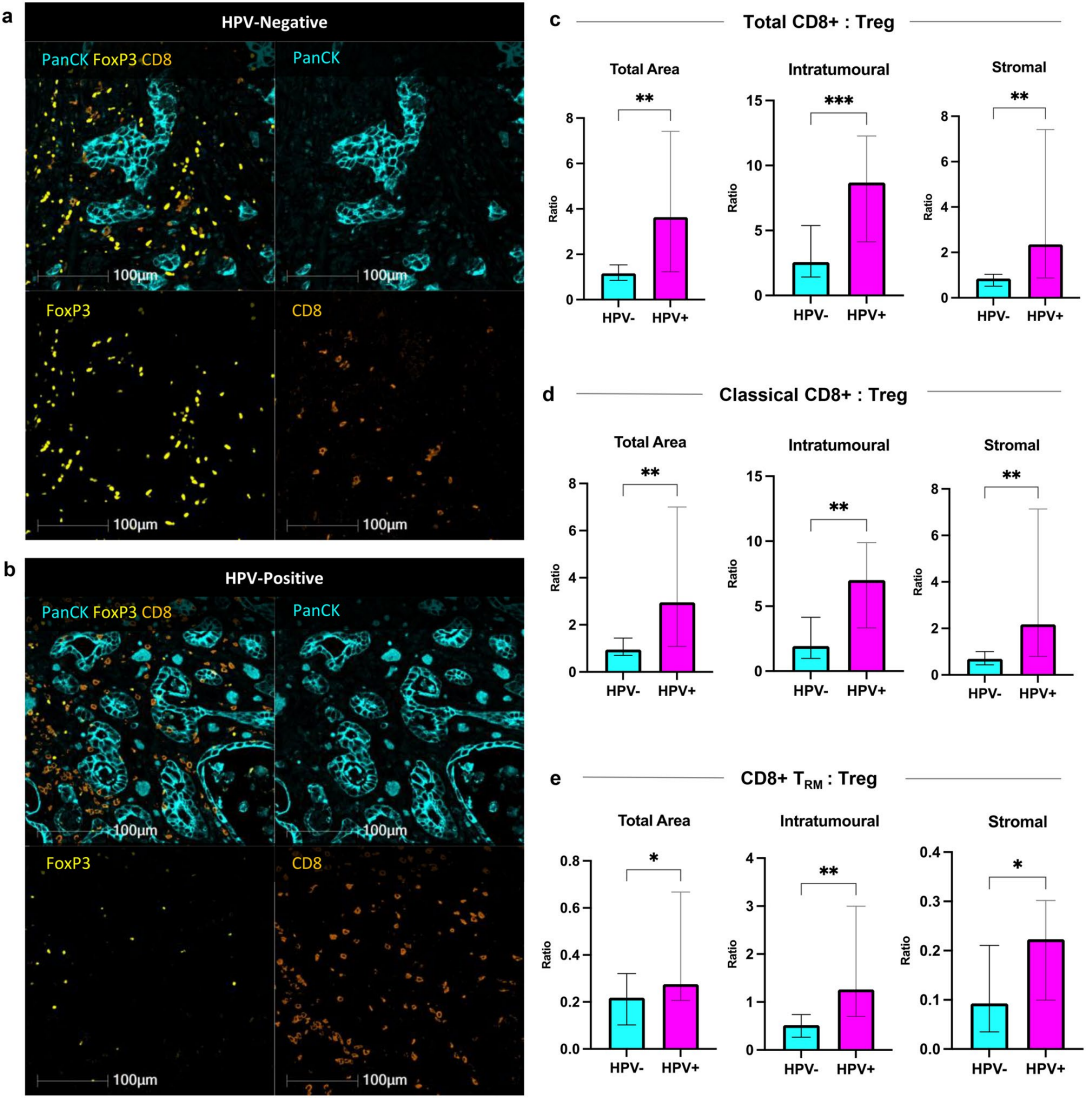
CD8+ T-cell subsets to Treg ratios HPV-negative and HPV-positive OAC. Representative multiplex immunofluorescence images of (**a**) HPV-negative and (**b**) HPV-positive OAC. A lower ratio of CD8+ (orange) T-cells to FoxP3+ Treg cells (yellow) is present in HPV-negative OAC compared to HPV-positive OAC. PanCK (cyan) identifies tumour cells (PanCK; cyan). Scale bar 100 μm. Representative images are zoomed areas within a 20x objective. (**c**) The ratio of total CD8+ T-cells to Treg densities. (**d**) The ratio of classical CD8+ T-cells to Treg densities. (**e**) The ratio of CD8+ T_RM_ T-cells to Treg densities. Unpaired nonparametric mann-whitney U tests were performed on (**c**)-(**e**). results are presented as median with 95% ci; *p < 0.05; **p < 0.01; ***p < 0.001

Given the cytotoxic nature of CD8+ T-cells and the suppressive function of Tregs, the CD8+:Treg ratio has been investigated as a prognostic indicator in a range of cancers [23–25], including oesophageal SCC [26]. Therefore, we examined the ratio of total CD8+, classical CD8+ and CD8+ T_RM_ T-cells to Tregs in the total, intratumoural and stromal areas (Fig. 4a–e). The ratios of total CD8+ T-cells (classical and T_RM_ CD8+ T-cell subsets) to Treg were lower in the HPV-negative group across the total (1.2 vs 3.6; *p* = 0.006), intratumoural (2.6 vs 8.7; *p* = 0.001) and stromal (0.9 vs 2.4; *p* = 0.004) areas (Fig. 4c). The median classical CD8+:Treg ratio was lower in the HPV-negative group as compared to the HPV-positive group: 1.0 vs 3.0 in the total area (*p* = 0.008), 1.9 vs 7.0 in the tumour (*p* = 0.003) and 0.7 vs 2.0 in the stroma (*p* = 0.004) (Fig. 4d). CD8+ T_RM_:Treg ratios were lower in the HPV-negative cohort compared to HPV-positive cohorts across all histological areas (Fig. 4e).

### Infiltration capacities of T-cell subsets towards tumour are similar

**Fig. 5:**
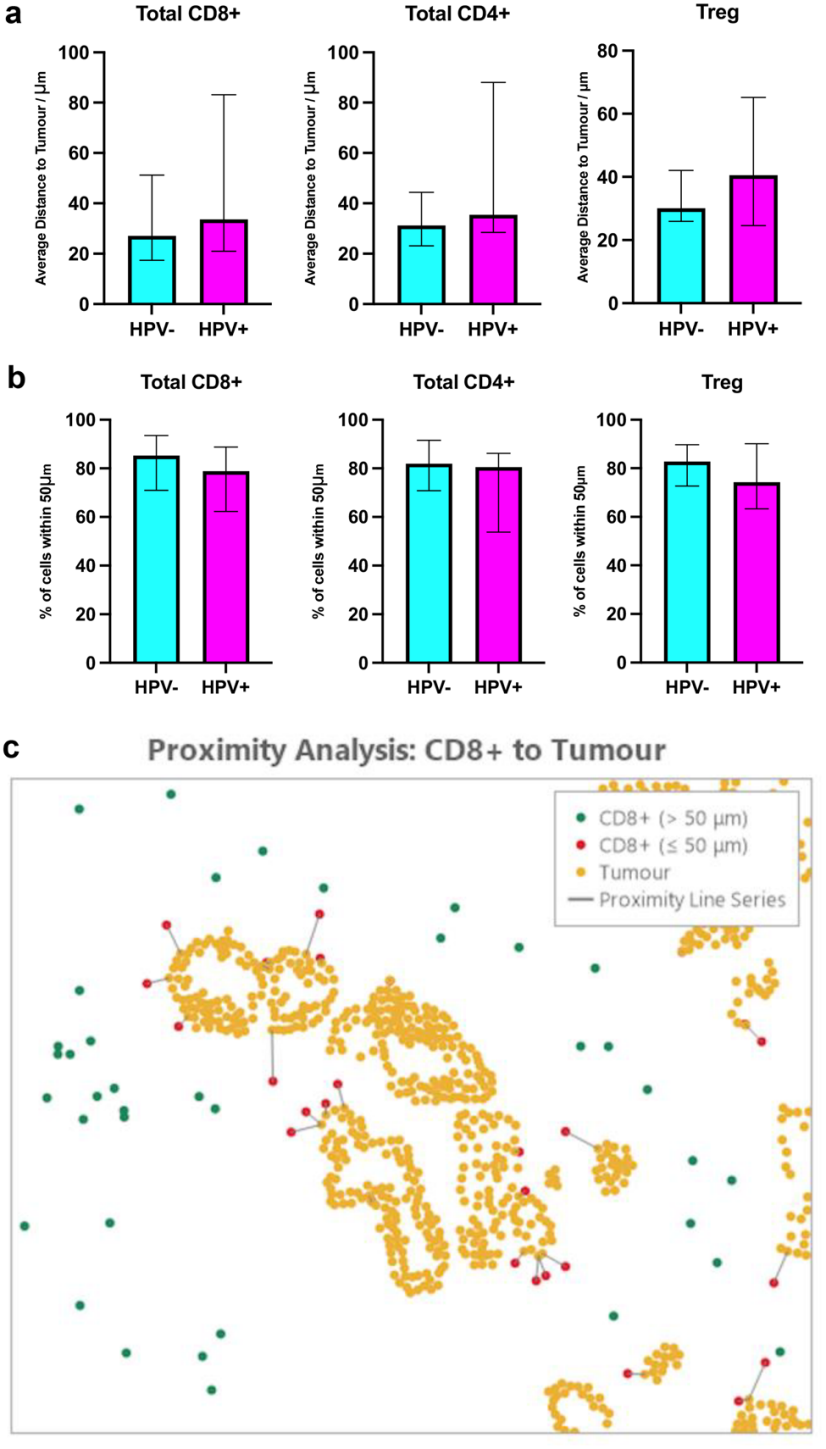
Infiltration capacities of T-cell subsets to tumour cells within OAC. (**a**) Average distances of total CD8+ T-cells, total CD4+ T-cells and Treg to a tumour cell. (**b**) Percentage of total CD8+ T-cells, total CD4+ T-cells and Treg within 50 μm of a tumour cell. (**c**) A schematic spatial plot of CD8+ T-cells showing CD8+ T-cells outside 50 μm range of tumour cells (Green); CD8+ T-cells within 50 μm (Red); Tumour cells (PanCK+; yellow). Results are presented as median with 95% ci. Unpaired nonparametric mann-whitney U tests were performed on (**a**)-(**b**); non-significant *p* < 0.05

We investigated the infiltration capacities of total CD8+ and CD4+ T-cells towards tumour region. Tregs were also explored given their differential proportions within the total, intratumoural and stromal areas across groups. Distances of total CD8+ T-cells (27.1 vs 33.6 μm; *p* = 0.098), total CD4+ T-cells (31.2 vs 35.5 μm; *p* = 0.225) and Tregs (30.1 vs 40.6 μm; *p* = 0.383) to a tumour cell were not statistically different between HPV-negative and HPV-positive OAC (Fig. 5a). The proportion of each T-cell subset infiltrating into the 50 μm radii of a tumour cell was not significant between the two HPV-negative and HPV-positive cohorts at 85.3 vs 78.9% (*p* = 0.250) for total CD8+ T-cells, 81.9 vs 80.6% (*p* = 0.250) for total CD4+ T-cells and 82.8 vs 74.3% (*p* = 0.511) for Treg respectively (Fig. 5b).

We further studied the spatial distribution by providing descriptive data only on the proportion of T-cells present within 50 μm at 10 μm intervals from a tumour cell. Interestingly, in both HPV-negative and HPV-positive TME, the proportion of cytotoxic Total CD8+ T-cells were highest within 0–10 μm and decreased in each category further away from the tumour (Supplementary Figure 4a). This contrasts to total CD4+ T-cells and FoxP3+ Tregs, both of which were highest in the 10–20 μm category (Supplementary Figure 4b-c), thus residing further away from tumour cells compared to total CD8+ T-cells.

### T-cell parameters that differ with HPV status correlate with overall survival

**Fig. 6:**
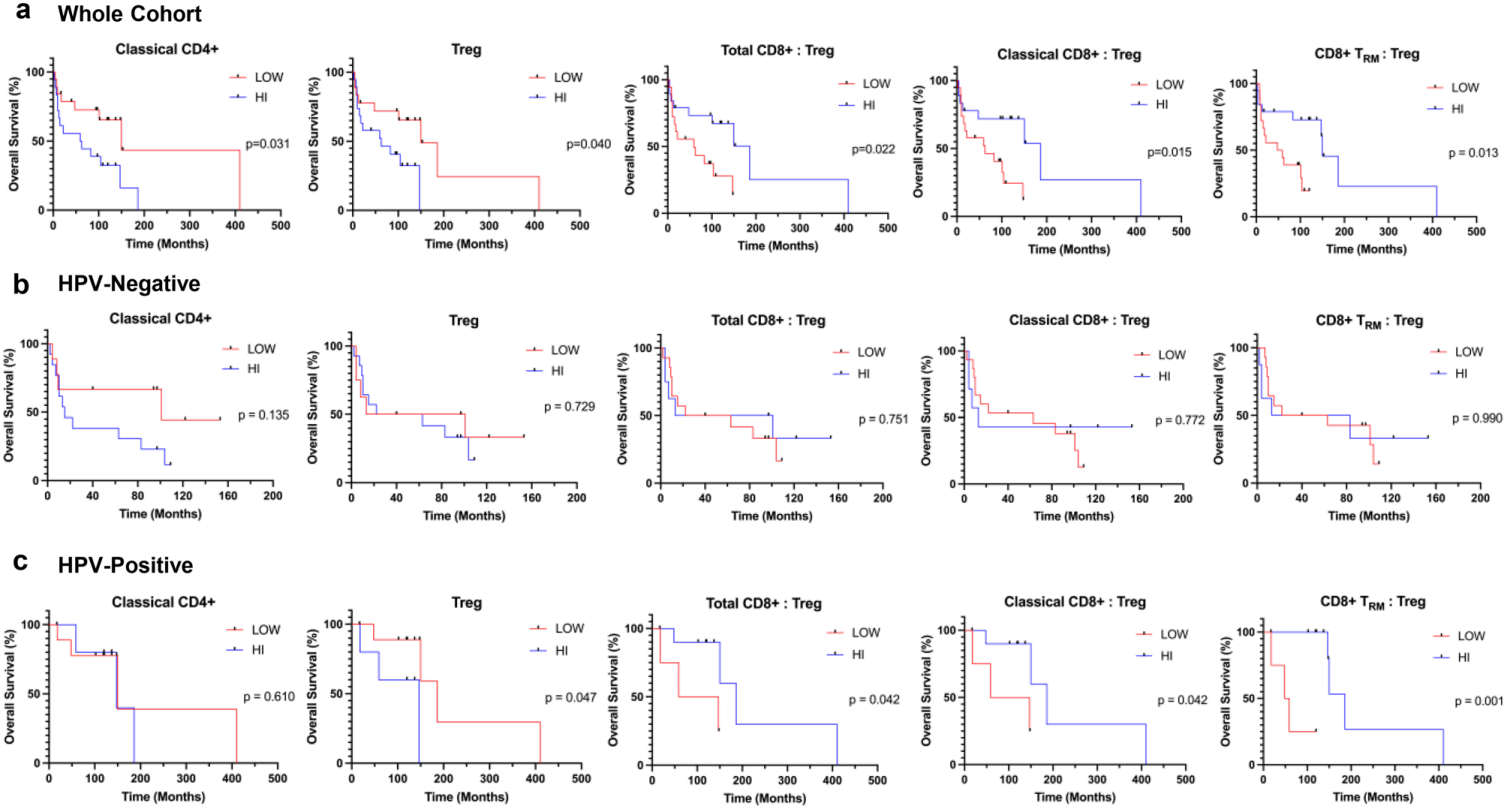
Relationship between T-cell subsets within total OAC tissue and patient overall survival. (**a**) OS of the total OAC cohort (n=37) according to high and low groups of classical CD4+ T-cell density (median cut-off: 1745/mm2); Treg density (median cut-off: 86.0/mm2); total CD8+:Treg ratio (median cut-off: 1.28); classical CD8+:Treg ratio (median cut-off: 1.09); CD8+ T_RM_:Treg ratio (median cut-off: 0.24). Kaplan-Meier survival curves of OS stratified into (**b**) HPV-negative (n=22) and (**c**) HPV-positive (n=15) groups. The same median cut-off was applied as the corresponding T-cell parameter in (**a**). Log-rank Mantel-Cox test p-value indicated on graphs; p<0.05 was considered statistically significant

The HPV-positive OAC group had a median OS of 121 months (range 17–410 months) as compared to 31 months (range 2–153 months) in the HPV-negative OAC group (*p* = 0.001). We investigated whether the proportion of major T-cell subsets in the total tissue area i.e. total CD4+, classical CD4+, Treg, tissue-resident CD4+, tissue-resident Treg, total CD8+, classical CD8+ and CD8+ T_RM,_ correlated with OS (Fig. 6a; Supplementary Figure S5). Across all patients, increased OS was associated with low classical CD4+ T-cells (150 vs 61 months; *p* = 0.03). Individuals with low Treg numbers were associated with longer median OS (150 vs 63 months; *p* = 0.04). High ratios of total CD8+:Treg (186 vs 59 months; *p* = 0.022), classical CD8+:Treg (186 vs 63 months; *p* = 0.015) and CD8+ T_RM_:Treg (150 vs 54 months; *p* = 0.013) were associated with significantly longer OS. Other major T-cell subsets showed no differences in OS between high and low groups (Supplementary Figure S5). Interestingly, T-cell parameters with a significant impact on patient OS corresponded to the densities that significantly differed between HPV-positive and HPV-negative groups (Fig. 2; 4).

Given the observed differences in classical CD4+ T-cell and Treg densities, as well as ratios of total CD8+:Treg, classical CD8+:Treg and CD8+ T_RM_:Treg, Kaplan-Meier survival curves were stratified by HPV status to explore any differential impact between these subgroups (Fig. 6b–c). The median cut-off value for each T-cell parameter that was derived from the total cohort was applied to each HPV-negative and HPV-positive group. For Treg and CD8:Treg ratios, HPV-negative cohorts did not show any differences in survival between low and high groups. Conversely, HPV-positive patients with high Treg, low total CD8+:Treg, classical CD8+:Treg and CD8+ T_RM_:Treg ratios were consistently associated with higher mortality (Fig. 6c). High and low groups of classical CD4+ T-cells did not show any differences in patient survival in both HPV-negative and HPV-positive groups.

### The total CD8+:Treg ratio is an independent predictor of patient prognosis

Given that low Treg density and high CD8+:Treg ratios were associated with better OS in all patients and in the HPV-positive group, we further evaluated the prognostic value of these measures through Cox regression hazards modelling to investigate whether they were statistically independent predictors of patient outcome. Univariate analyses of the clinicopathological characteristics from Table 1 revealed that no lymph node involvement, no metastasis and HPV-positivity were associated with better OS (Table 2). In a multivariate analysis (MVA; Table 2) adjusting for these factors, a low total CD8:Treg ratio was an independent predictor of poorer OS (HR 3.06; 95% CI 1.05–10.27; *p* = 0.050). There was a trend suggesting that low Treg density may be associated with improved overall survival (OS) (HR 0.33; 95% CI 0.10–1.01; *p* = 0.060). Of note, HPV status demonstrated independent survival benefits in MVA models with Treg, total CD8+:Treg, and classical CD8+:Treg ratios.

**Table 2:** Univariate and multivariate analyses of variables associated with overall survival (OS)

Variables	HR	95% CI	*p* value
**Univariate Analysis**			
Age	0.98	0.95–1.02	0.364
Sex (Male vs Female)	0.99	0.35–3.50	0.983
T stage (Low vs High)	0.41	0.16–1.02	0.054
N stage (Neg vs Pos)	0.27	0.11–0.65	0.004*
M stage (Neg vs Pos)	0.24	0.09–0.64	0.004*
HPV Status (HPV+ vs HPV-)	0.26	0.08–0.69	0.011*
**MVA with Treg**			
Age	0.95	0.89–1.00	0.071
Sex (Male vs Female)	0.72	0.15–3.72	0.677
T stage (Low vs High)	0.83	0.23–2.93	0.772
N stage (Neg vs Pos)	0.54	0.16–1.78	0.317
M stage (Neg vs Pos)	0.33	0.09–1.14	0.078
HPV Status (HPV+ vs HPV-)	0.23	0.06–0.71	0.017*
Treg (Low vs High)	0.33	0.10–1.01	0.060
**MVA with Total CD8+:Treg**			
Age	0.96	0.91–1.02	0.176
Sex (Male vs Female)	0.86	0.18–4.46	0.846
T stage (Low vs High)	0.73	0.21–2.52	0.615
N stage (Neg vs Pos)	0.54	0.15–1.75	0.309
M stage (Neg vs Pos)	0.29	0.08–1.01	0.048*
HPV Status (HPV+ vs HPV-)	0.26	0.07–0.85	0.032*
Total CD8+:Treg (Low vs High)	3.06	1.05–10.27	0.050*
**MVA with Classical CD8+:Treg**			
Age	0.97	0.92–1.02	0.210
Sex (Male vs Female)	1.38	0.30–7.40	0.695
T stage (Low vs High)	0.70	0.19–2.61	0.596
N stage (Neg vs Pos)	0.63	0.17–2.09	0.463
M stage (Neg vs Pos)	0.37	0.10–1.28	0.110
HPV Status (HPV+ vs HPV-)	0.27	0.08–0.87	0.034*
Classical CD8+:Treg (Low vs High)	2.19	0.74–7.34	0.170
**MVA with CD8+ T**_**RM**_**:Treg**			
Age	0.96	0.91–1.01	0.141
Sex (Male vs Female)	1.13	0.23–6.18	0.886
T stage (Low vs High)	0.89	0.22–3.75	0.869
N stage (Neg vs Pos)	0.50	0.12–1.80	0.310
M stage (Neg vs Pos)	0.45	0.12–1.68	0.216
HPV Status (HPV+ vs HPV-)	0.35	0.09–1.14	0.090
CD8+ T_RM_:Treg (Low vs High)	3.05	0.89–12.73	0.093

HR, hazard ratio; CI, confidence interval; T, tumour; N, lymph node; M, metastasis; MVA, Multivariate analysis. *p < 0.05 was considered significant

## Discussion

To our knowledge, this is the first study to report a detailed mIF analysis of T-cell subsets in the HPV-negative and HPV-positive TME of OAC. We found a higher number of FoxP3+ Tregs and lower ratios of CD8+ T-cells to Tregs in HPV-negative OAC patients compared to HPV-positive counterparts. This suggests an immunoregulatory TME in HPV-negative OAC. Our findings align with current evidence of divergent pathogeneses of HPV-negative and HPV-positive OAC. Classically, both OAC and its precursor Barrett’s oesophagus harbour high frequencies of mutations in the tumour repressor gene *TP53* [27]. While 50% of HPV-negative cases were found to carry mutations of *TP53* by whole genome sequencing, no mutations were detected in any HPV-positive cases [28]. This is consistent with the pathology of HPV-driven cancers, where the oncoprotein E6 downregulates but preserves wildtype *TP53* [29]. Mutant *TP53* has been associated with increased Treg and decreased CD8+ T-cells in both number and cytotoxic function in gastric adenocarcinoma, supporting the immune profile of HPV-negative OAC observed in our study [30]. Additionally, an HPV-negative OAC TME is more prone to the Warburg effect [31], which is an accumulation of lactic acid due to increased metabolic demand of the tumour. Studies show that this acidity reduces CD8+ T-cell number and activity [31, 32], while Tregs consume lactic acid, allowing for effective cell proliferation [33].

Our findings of T-cell subset densities contrast with those reported in HNSCC, where the TME of HPV-negative and HPV-positive cases have been extensively profiled. Firstly, the characterisation of Treg numbers in HNSCC remains inconsistent across studies. Whilst some report significantly higher Treg in HPV-positive HNSCC [17, 34–38], others have observed no notable differences [39, 40], and one study even reported a lower proportion of Treg in HPV-positive cases [41]. Discordance in staining between FoxP3 antibody clones has been postulated to underlie the heterogeneity in results [42]. Notably, studies reporting increased FoxP3+ Tregs in HPV-positive cohorts also identified a higher proportion of total CD4+ T-cells, suggesting that the Treg increases may reflect broader CD4+ T-cell infiltration rather than a selective expansion of the Treg subset [36, 38]. This interpretation is supported by Hur et al., who reported a lower proportion of Tregs in HPV-positive HNSCC, together with a reduced total CD4+ T-cells [42]. Concordantly, we found elevated Treg in the HPV-negative cohort, along with higher total and classical CD4+ T-cell densities in the stromal areas of HPV-negative OAC. However, our study shows significant results in both stromal and intratumoural regions, indicating that the increase in intratumoural Tregs is not simply due to overall CD4+ T-cell infiltration. This supports the previously discussed Warburg effect, as intratumoural Tregs have been shown to exhibit greater lactate uptake compared to Tregs in the peripheral TME [33].

In contrast to our OAC findings, several studies investigating HNSCC have reported a higher proportion of total CD8+ T-cells [17, 31, 34, 35, 37, 43] and CD8+ T_RM_ T-cells [17, 44] in the HPV-positive TME compared to HPV-negative cases. In a retrospective cross-sectional study, Tosi et al. employed a mIF workflow comparable to that used in the present study, and reported increased densities of total CD8+ T-cells, along with closer proximity to tumour cells in HPV-positive HNSCC [17]. These results contrast with our study which did not show significant differences in densities and infiltration capacities of CD8+ T-cells. Despite these differences, a higher intratumoural CD8+:FoxP3 ratio has been reported in HPV-positive HNSCC, which is concordant with our study [36]. Therefore, the overall balance of the TME appears to trend towards cytotoxicity in HPV-positive cases and immunoregulation in HPV-negative cases and this may highlight the roles of both CD8+ T-cells and Tregs in HPV-driven cancers. However, it is important to note that published studies have described distinct TMEs between HNSCC and OAC [45]. Despite their close anatomical proximity, HNSCC is a well-established HPV-associated cancer, whereas the casual role of HPV in OAC remains unconfirmed. Thus, further studies comparing HPV-associated and HPV-independent OAC are necessary to validate our findings.

Our data demonstrated that T-cell parameters that varied between HPV-negative and HPV-positive cohorts were significantly related to clinical outcomes, potentially explaining favourable patient outcomes of HPV-positive OAC. Elevated classical CD4+ T-cells, high Treg and low CD8+:Treg ratios correlated with shorter OS when assessed in the total cohort. This suggests that high CD4+ T-cell numbers may primarily be a product of Tregs, a subset of classical CD4+ T-cells. A meta-analysis suggests that high Treg is associated with poorer patient outcome in various cancer types [46], corresponding to its suppressive action and our findings. This association of immunosuppressed TME and adverse clinical outcome aligns with previous studies on OAC. Witt *et al.* found that high FoxP3+ density was an independent predictor of poor patient outcome in oesophageal cancers, the majority of which were adenocarcinoma (HR 2.18, 95% CI 1.25–3.80, *p* < 0.01) [47]. Conversely, unlike our findings which showed no significant association of CD8+ T-cell subsets with OS, CD8+ T-cell density and their proximity to tumour cells have positively correlated with prolonged survival in other studies of OAC [14, 48]. While CD8+ T-cells alone may have a protective role in OAC, differences in survival outcomes based on HPV status are more likely driven by variations in Treg density and CD8+:Treg ratios.

When the correlation between T-cell levels and patient outcome was further stratified by HPV status, HPV-positive OAC patients with high Treg density and low CD8+:Treg ratios had lower OS. Strikingly, this is analogous to HNSCC, where high total T-cells in situ were predictive of improved survival. However, HPV-positive patients with low T-cells exhibited poor survival outcomes, resembling those in HPV-negative patients [17, 38, 43]. Hence, the anti-inflammatory environment, characterised by low T-cell infiltration in HNSCC and a relatively immunoregulated TME in OAC of our study, may account for the survival disadvantage seen in HPV-negative patients as well as in a subgroup of HPV-positive patients exhibiting a similar immune profile. The prognostic value of T-cells is further supported by our MVA which revealed total CD8+:Treg ratio to be an independent predictor of OS. Low Treg density alone showed a trend towards better survival but did not reach significance and this may be a product of our small sample size. Nasman et al. reported a high CD8+:FoxP3 ratio to be associated with a positive outcome in HNSCC independent of HPV status [34]. This supports our study findings that the high CD8+:Treg ratio reflects the relative level of cytotoxicity and could serve as a valuable biomarker [23–26].

A strength of this work includes the methodology of mIF microscopy which offers detection of specific T-cell subsets by co-localising multiple markers, and automated analysis resulted in less inter-observer variations and subsequently more reproducible data [49]. Additionally, we analysed the whole ROI identified by the pathologist, instead of constructing tissue microarrays or selecting regions which can add bias by identifying immune ‘hot’ or ‘cold’ areas. However, we acknowledge that this is a retrospective study involving a small sample size (*n* = 37), and larger studies are required to validate our results. Finally, the median cut-off from an internal cohort was applied to assess differences in OS. External validation in a larger cohort and determining the optimal cut-off value are necessary to translate this work into clinical utility as a predictive biomarker.

Future work should investigate other components that dynamically shape the TME between HPV-positive and HPV-negative OAC such as inhibitory checkpoints important for T-cell exhaustion e.g. Programmed cell death protein-1 (PD-1), B-cells, myeloid cells, tumour-associated macrophages and differential cytokines profiles [45]. Technologies such as single-cell spatial transcriptomics and TME-targeted mIF panels may help to delineate the key immune differences. Additionally, modulating the immunosuppressive TME has the potential to improve the efficacy of therapeutic vaccination in this subset of virus-associated cancers. Indeed, preliminary work investigating a phase I trial of vaccination against p53 showed efficacy in decreasing Treg levels in HNSCC [50].

## Conclusion

In conclusion, this work demonstrates for the first time that HPV-negative OAC is associated with an immunoregulatory TME, which is associated with shorter OS. Multivariate models identified a high total CD8+:Treg ratio, together with HPV presence and absent metastasis, to be independent predictors of prolonged OS. Further validation would support the possibility that both HPV positivity and CD8+:Treg ratio could be useful tools for future clinical decision-making.

## Electronic supplementary material

Below is the link to the electronic supplementary material.

Supplementary material 1

## Data Availability

The datasets used and analysed during the current study are available from the corresponding authors (SR and SS) on reasonable request.

## Abbreviations

SCC: Squamous cell carcinoma
OAC: Oesophageal adenocarcinoma
GORD: Gastro-oesophageal reflux disease
HPV: Human papillomavirus
HNSCC: Head and Neck Squamous Cell Carcinoma
DFS: Disease-free survival
OS: Overall survival
TME: Tumour microenvironment
ICI: Immune checkpoint inhibitor
FFPE: Formalin-fixed paraffin-embedded
PCR: Polymerase chain reaction
mIF: Multiplex immunofluorescence
ROI: Regions of interest
Treg: Regulatory T-cells
MVA: Multivariate analysis
HR: Hazard ratio
