# Supplementary material for: HPV status shapes T-cell immunoprofiles in oesophageal adenocarcinoma: high regulatory T cell infiltration predicts poor prognosis
Source: J Transl Med. 2025 Nov 27;24:12. doi: 10.1186/s12967-025-07482-3 (PMC12763987; doi:10.1186/s12967-025-07482-3)
Supplement: Supplementary file 1 — Supplementary material 1 [file 12967_2025_7482_MOESM1_ESM.pdf]

Supplementary Tables

Supplementary Table S1. Sequences and positions of PCR and real-time primers

	Forward Primers (5'-3')	Position	Reverse Primers (5'-3')	Position
**HPV16 HQ644299**	AAC CGA AAT CGG TTG AAC CG	34-53	TGA TGT GTA TGT AGA CAC AGA C	3972-3951
	TGC ACC AAA AGA GAA CTG CA	84-103	GCG GAC CTA TTA ATA GGC AGA C	3943-3922
	(E6F) GAG AAC TGC AAT GTT TCA GGA CC	94-116	(E6R) TGT ATA GTT GTT TGC AGC TCT GT	174-152
	(E2F) AAC GAA GTA TCC TCT CCT GAA AT	3362-3384	(E2R) CCA AGG CGA CGG CTT TG	3443-3427
**HPV 18 GQ180792**	GGA GTA ACC GAA AAC GGT	36-53	CAT ACA TGC ATA CAC AAA AGC	3981-3961
	ATG TGA GAA ACA CAC CAC AAT A	81-102	GAT AAC ATA TTG GTA CTA CAG CAT	3943-3920
	(E6F) GCT TTG AGG ATC CAA CAC G	112-130	(E6R) TTC TAT GTC TTG CAG TGA AGT GTT C	167-192
	(E2F) GAA CAC AGG TAC GTG GGA AGT	3368-3688	(E2R) CGT GTC GTC ACT GGT ACT GC	3449-3430
**Human albumin gene**	ACC ATG CTT TTC AGC TCT GG		TCT GCA TGG AAG GTG AAT GT	

Supplementary Table S2. Nested PCR and Viral load of HPV-positive patients

Deidentified Patient No.	HPV Gene	Viral load (copies/1000cells)
**1**	E	69.92
**2**	L	4.85
**3**	L	28.33
**4**	L	0.06
**5**	L	4.18
**6**	L	5.81
**7**	E	14.98
**8**	E	115.03
**9**	E	1.38
**10**	E	17.24
**11**	L	34.43
**12**	L	58.64
**13**	E	28.48
**14**	L	1.47
**15**	E	0.24

Supplementary Figures

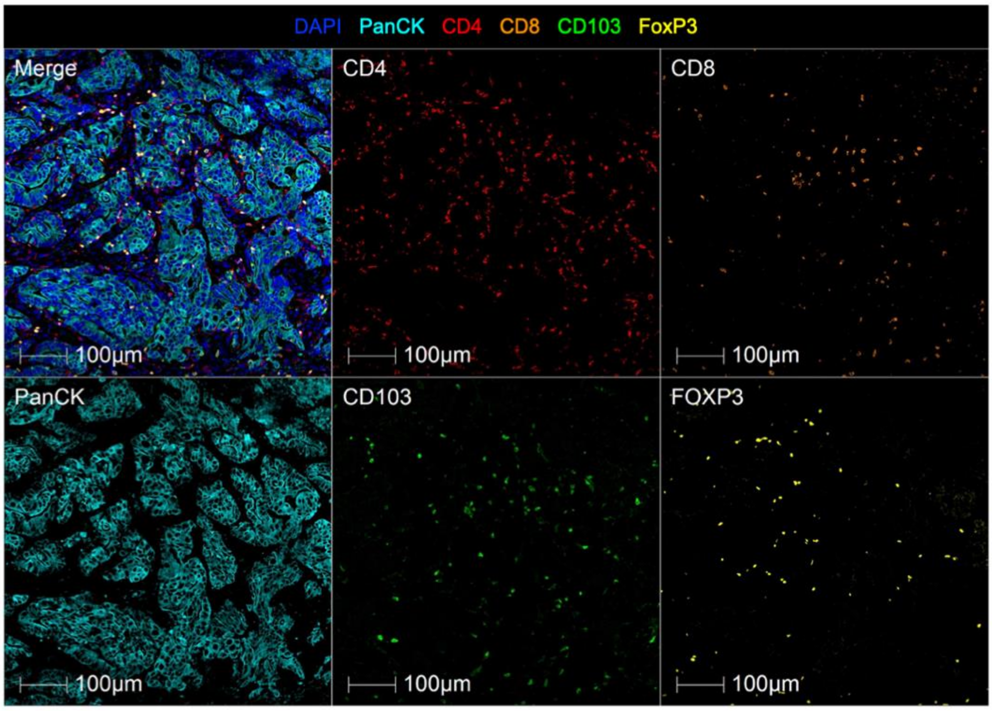

Supplementary Figure S1. Representative image of a multiplex immunofluorescence panel. DAPI (blue), PanCK (cyan), CD4 (red), CD8 (orange), CD103 (green) and FoxP3 (yellow). Scale bar 100µm; Representative images are zoomed areas within a 20x objective.

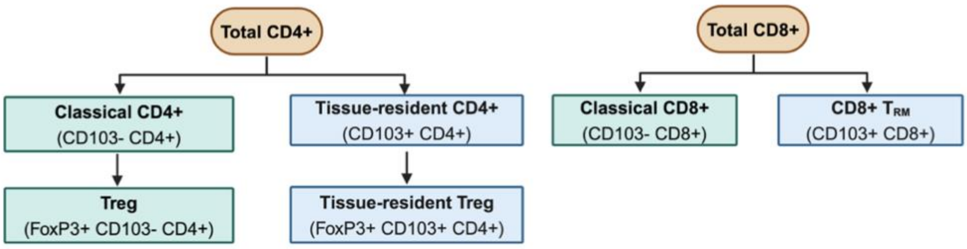

Supplementary Figure S2. Phenotypic characterisation of CD4+ and CD8+ T-cell subsets of interest. T-regulatory cell (Treg); Tissue-resident memory (T_{RM}) T-cell. Created with BioRender.com.

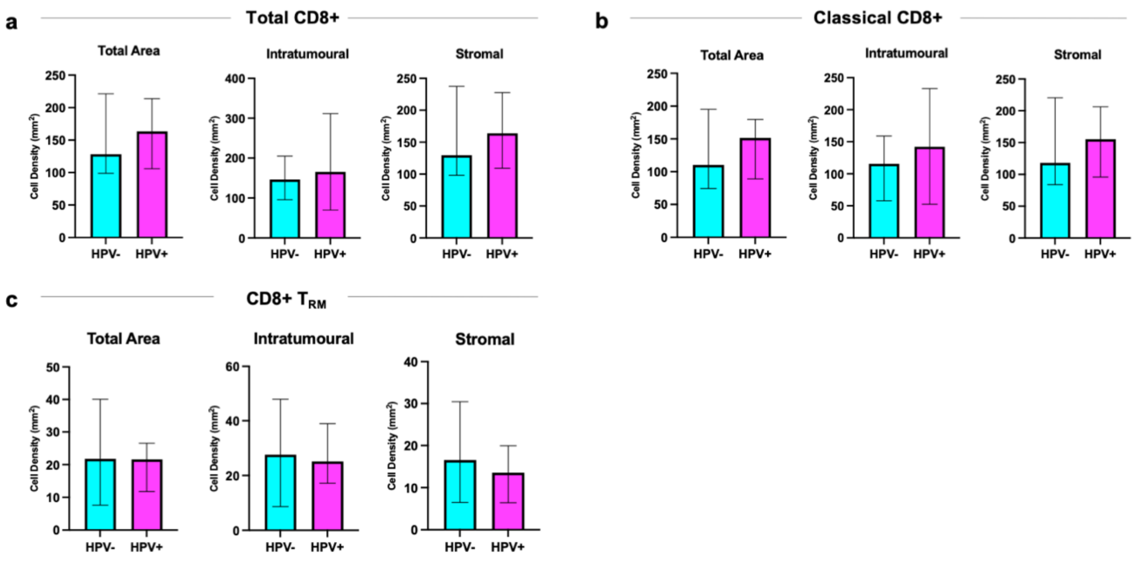

Supplementary Figure S3. CD8+ T-cell densities HPV-negative and HPV-positive OAC. (a) Total CD8+ T-cells. **(b)** Classical CD8+ T-cells. **(c)** CD8+ TRM T-cells. Unpaired nonparametric Mann-Whitney U tests were performed on **(a)-(c).** Results are presented as median with 95% CI; non-significant results $p>0.05$.

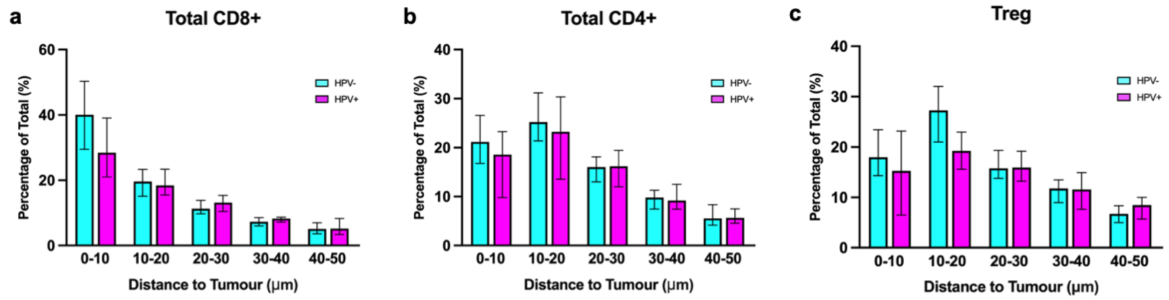

Supplementary Figure S4. Spatial distribution of T-cell subsets within 50µm of OAC Tumour cells. Percentage detected within 50µm of a tumour cell at 10µm increments for **(a)** Total CD8+ T-cells; **(b)** Total CD4+ T-cells and **(c)** Treg. Results are presented as median with 95% CI. No statistical test was applied on **(a)-(c)**.

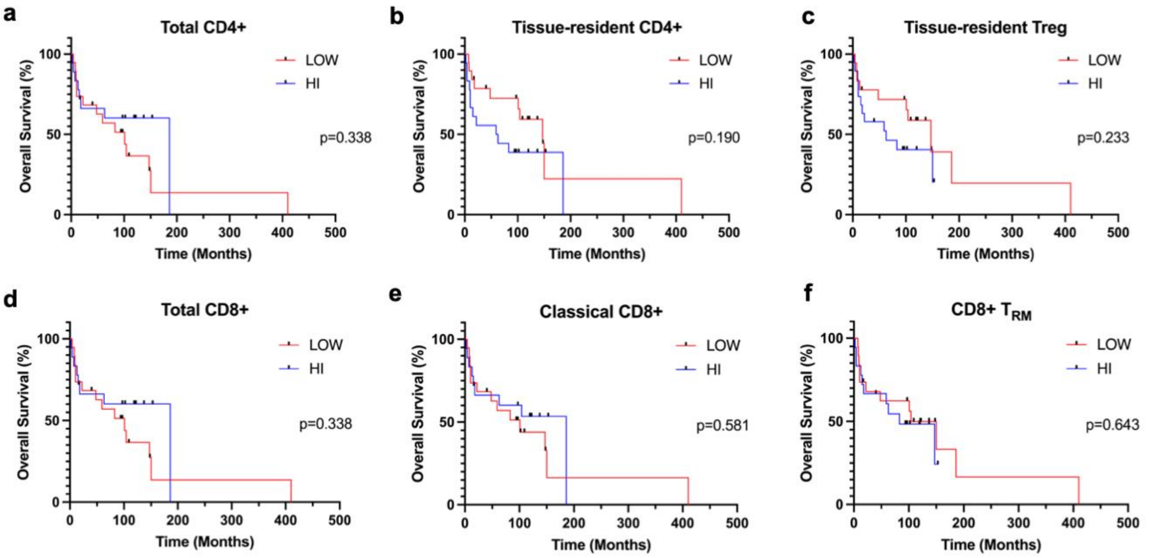

Supplementary Figure S5. Kaplan-Meier curves demonstrating the relationship between T-cell subsets within total OAC tissue and patient survival. Curves show OSof the total OAC cohort (n=37) according to high and low groups of **(a)** Total CD4+ T-cell density (median cut-off: 1779/mm2); **(b)** Tissue-resident CD4+ T-cell density (median cut-off: 34.2/mm2); **(c)** Tissue-resident Treg density (median cut-off: 0.40/mm2); **(d)** Total CD8+ T-cell density (median cut-off: 140.8/mm2); **(e)** Classical CD8+ T-cell density (median cut-off: 123.3/mm2); **(f)** CD8+ T_{RM} T-cell density (median cut-off: 21.6/mm2). Log-rank Mantel-Cox test p-value indicated on graphs; p<0.05 was considered statistically significant.
